# Nitrogen Oxides Mitigation Efficiency of Cementitious Materials Incorporated with TiO_2_

**DOI:** 10.3390/ma11060877

**Published:** 2018-05-24

**Authors:** Inkyu Rhee, Jun-Seok Lee, Jong Beom Kim, Jong-Ho Kim

**Affiliations:** 1 Department of Civil Engineering, Chonnam National University, Gwangju 61186, Korea; 2 Bio-Housing Institute, Chonnam National University, Gwangju 61186, Korea; hertz002@nate.com; 3 School of Chemical Engineering, Chonnam National University, Gwangju 61186, Korea; mask-k@daum.net (J.B.K.); jonghkim@jnu.ac.kr (J.-H.K.)

**Keywords:** titanium dioxide, cement paste, mortar, compressive strength, nitrogen oxides, apparent density

## Abstract

We explored the photocatalytic capacities of cementitious materials (cement paste and mortar) incorporating titanium dioxide (TiO_2_). P-25 is a commercial TiO_2_ preparation which, if incorporated into large civil buildings, is extremely expensive. It is essential to produce low-cost TiO_2_. A cheap anatase form of TiO_2_ powder, NP-400, manufactured under relatively low burning temperature, was considered in this paper. Addition of NP-400 to 0, 5, 10, and 20 wt % did not significantly affect the compressive strengths of mortar or cement paste. However, the compressive strengths of P-25-containing specimens were more consistent than those of NP-400-containing materials. The nitrogen oxide (NO) removal efficiencies by mortar with 5 and 10 wt % TiO_2_ were similar at ca. 14–16%; the removal efficiency by mortar with 20 wt % NP-400 was ca. 70%. Although the NP-400 cluster size was almost halved by ultrasonication, NO removal efficiency was not enhanced. Removal was enhanced by the presence of accessible surface area: NP-400 dispersed in these surfaces readily adsorbed NO, aided by the large surface areas of the top and bottom faces. Scanning electron microscopy coupled with energy dispersive X-ray analysis (SEM–EDX) confirmed that NP-400 tended to sink when added to cement, fine aggregates, and water because the true densities of P-25, NP-400, and cement powder differed (3.41, 3.70, and 3.15 g/mL). The true density of NP-400 was thus the highest of all ingredients. The relatively low apparent density of P-25 compared to that of NP-400 was associated with a more bulky distribution of P-25 within cementitious materials. Nevertheless, NP-400 could be a viable alternative to the definitive product, P-25.

## 1. Introduction

Recent rapid climate change has greatly affected the air quality of the Korean peninsula. Micro-sized industrial pollutants increasingly produced by neighboring countries are borne to Korea by wind year-round. Also, internal factors, such as power plants burning fossil fuels and diesel-powered vehicles, have significant environmental effects. Microdusts are readily adsorbed by the outer walls of civil infrastructure and residential facilities, affecting public health and the quality of city life [1,2,3]. In Japan, Italy, Belgium, China, and the United States, photocatalytic titanium dioxide (TiO_2_) is added to sidewalk blocks, precast external building material, and the walls of large buildings such as theaters and stadiums; TiO_2_ exhibits anti-fouling, deodorization, and air purification properties. Commercial TiO_2_ is used in the construction of such conventional structures [4,5,6,7,8,9,10]. The most common crystalline phases of TiO_2_ are rutile, anatase and brookite. Among these phases, the anatase is the most widely used for photocatalytic reactions because of its large surface area, stability and higher activity compared to the rutile [11]. When TiO_2_ is exposed to ultraviolet (UV) light, it can absorb photon energy equal to or larger than its band gap, promoting electrons to jump from the valence band to the conduction band. The activation of the electrons results in the generation of holes in the valence band. The electron-hole pairs may combine in a short time to initiate redox reactions depending on ambient conditions [12]. Optimal photocatalytic efficiency may be achieved from a mixture of anatase with a small percentage of rutile as electron hole recombination is prevented by the creation of energy wells which serve as an electron trap formed from the lower band gap of rutile, 3.0 eV against 3.2 eV for anatase [13]. Addition of TiO_2_ powder to cement mixes was found to significantly affect the hydration rate of cement and the properties of cement pastes and mortars. According to several authors [4,12,14,15,16], the early stage hydration rate and hydration degree of cement was significantly enhanced. Fluidity and strength at evening ages were found to decrease [17]. This leads the strength of cement mortar at early ages to increase a lot and the fluidity and strength at evening ages to decrease, obviously [17]. The workability and setting time of fresh mortar and concrete were decreased by increasing the content of TiO_2_ nanoparticles [12,14,15,16,17,18,19]. These aspects were consistently reported in even higher concentrations of TiO_2_ up to 15 wt % at the early stage of C_3_S hydration. The hydration product is formed on or near the surface of TiO_2_ particles, as well as on the C_3_S surface in 5, 10 and 15 wt % of TiO_2_ addition [14]. The compressive strength of cement mortar with 0–20 wt % of TiO_2_ addition are generally increased [18]. However, it is still controversial whether the added TiO_2_ particles have certain pozzolanic activity or they are only fine non-reactive fillers. It seems that TiO_2_ was inert and stable during the hydration process because the variation of relative mass ratios of TiO_2_ at different curing ages was very small [12]. Higher TiO_2_ concentration led to more TiO_2_ deposition on the surface of hydration products and fine aggregates and higher photocatalytic activity. Nevertheless, higher TiO_2_ concentration stirred up an agglomeration problem of TiO_2_, which led to more recombination of electron-holes and difficulties for TiO_2_ particles to be exposed to the photocatalytic reaction [20]. Comparing the nitrogen oxide (NO) degradation rates, the compositions in lower additions (0.5 and 1 wt %) exhibit high photocatalytic activity. Increasing TiO_2_ content from 2.5 to 5 wt % produced an increase in the NO degradation not sufficient to compensate for the decrease in mechanical strength of hardened cement mixes [21]. Research [22] on the different particle size effect on TiO_2_ in hardened cement: micro-sized TiO_2_ (avg. diameter—150 nm, SSA—8.7 m^2^/g) and nano-sized TiO_2_ (avg. diameter—18 nm, SSA—78.9 m^2^/g) was explored. Micro-sized TiO_2_ shows smaller and better dispersed agglomerates than nano-sized TiO_2_ even though the primary particle size (crystallite size) is bigger. Big particle agglomerate pores, small and highly dispersed agglomerates of micro-sized TiO_2_ offer a higher available surface area for adsorption and reaction of big molecules like Rhodamine B which hardly penetrate nano-sized TiO_2_ particle agglomerate pores. On the other hand, very small molecules like nitrogen oxides which can easily penetrate into nano-sized pores of agglomerates are better degraded by nano-sized TiO_2_. In this case, dispersion and the agglomerates porosity are not crucial; the available surface area is most likely to be due to the specific surface area determined by primary particle size [22]. Photocatalysis is a surface phenomenon, influenced by the chemistry of the immediate environment. The concrete surface must be engineered to maximise photocatalyst accessibility to reactants and activation. Catalyst surface area must be maximised for the target application; care must be taken to ensure particle dispersion is optimised. Agglomeration can block access to the internal surface, i.e., if pollutant molecule size is greater than pore entry diameter [23]. Photocatalytic experiments on the photocatalytic coatings on concrete and plaster substrates were carried out in two types (laminar flow, ideally-mixed flow) of flow reactors under real world conditions of temperature, relative humidity, irradiation intensity and pollutant concentrations [24,25]. However, TiO_2_ clusters sink slowly to the bottom of cementitious material prior to setting and hardening. This creates variations in TiO_2_ concentration between the top and bottom faces, especially during in situ construction. None of silica fumes, high-range water reducers, viscous agents, or blast furnace slag enhanced the dispersion characteristics of TiO_2_ clusters [26]. A combination of a foaming agent, a hardening accelerator, a viscous agent, and a small fine aggregate grain size aided TiO_2_ dispersion in cementitious materials but NO removal by the top surface was only minimally affected [26]. P-25 is a commercial TiO_2_ preparation which, if incorporated into large civil buildings, is extremely expensive. It is essential to produce low-cost TiO_2_, for example from the coagulant-containing sludge of wastewater treatment [27,28,29]. The NP-400 form of TiO_2_ manufactured in Korea is equivalent to P-25 in terms of mechanical and catalytic properties, but is only half the price. Here, we evaluated the compressive strengths and NO removal efficiencies of cement pastes/mortars mixed with NP-400.

## 2. Materials and Methods

### 2.1. Commercial Titanium Dioxides

**Figure 1:**
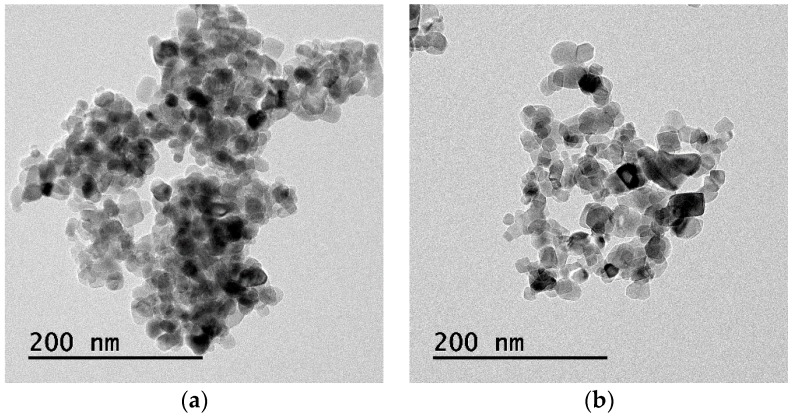
TiO_2_: transmission electron microscope (TEM) images of (**a**) NP-400, Bentech Frontier, Korea; and (**b**) P-25, Evonik, Germany.

**Figure 2:**
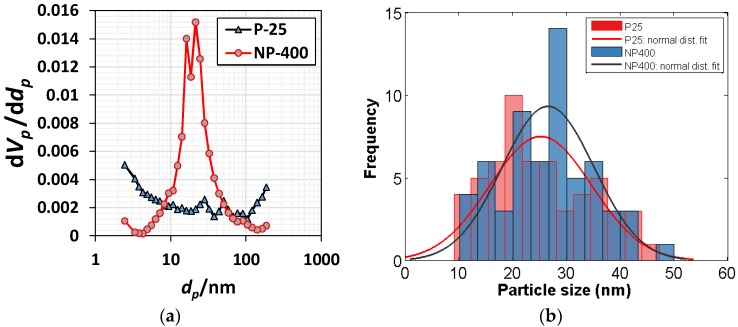
Pore distribution and particle size: (**a**) Barrett–Joyner–Halenda (BJH) plots to characterize the frequency distribution of pores; and (**b**) particle size distribution (nm) from TEM images.

**Figure 3:**
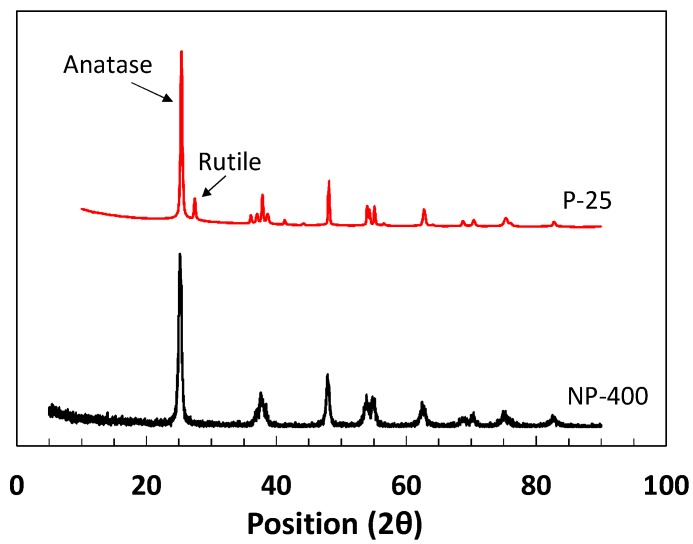
X-ray diffraction data for NP-400 and P-25 (Anatase: 2θ = 25.37°, Rutile: 27.41°).

Titanium chloride, TiCl_4_, is normally extracted from titanium precursor using hydrochloric acid. When water is added, hydrolysis produces titanium hydroxide, Ti(OH)_4_. After drying, the Ti(OH)_4_ powder is held in a vertical rotary kiln at 600 °C for 4–5 h; NP-400 collects in the bottom of the kiln. P-25 is relatively bulky and fluffy, because it is rapidly produced by direct spraying of TiCl_4_ into the kiln at 1000–1200 °C; the particles are minimally agglomerated. As the burn temperature is lower, NP-400 is anatase-like, thus unstable; P-25 is more stable. Normally, crystallinity is crucial in terms of a higher burn temperature. However, an unstable (amorphous) structure may be more photocatalytic after hydration within cementitious materials. In large-scale civil engineering projects, the price of TiO_2_ is critical. The cost of NP-400 is only 50% that of P-25, but the material performances are similar. Table 1 compares NP-400 and P-25. The apparent density of P-25 (in mixed rutile/anatase phases; Evonik, Essen, Germany) is 0.18 g/mL, whereas that of NP-400 (anatase phase; Bentech Frontier, Gwangju, Korea) is 0.45 g/mL. The volume occupied by P-25 is 2.5-fold that of NP-400 within the same mass. Figure 1 shows the transmission electron microscope (JEM-2100F, JEOL, Tokyo, Japan) images of NP-400 and P-25. Fifty TiO_2_ particles of each of NP-400 and P-25 were evaluated (Figure 2); the average particle sizes were similar. X-ray diffraction (PANanalytical X’Pert, Almelo, The Netherlands) data (Figure 3) revealed that the peak patterns at 25.36–25.38° are similar, being those of anatase-type powders. Slight differences in the peak intensities around 27.41° indicate that rutile-type powders are also present, especially in P-25. Normally, rutile-type powders are more crystalline than anatase-type powders, affecting burn temperatures, as mentioned above.

### 2.2. Cementitious Mixes Preparation

**Figure 4:**
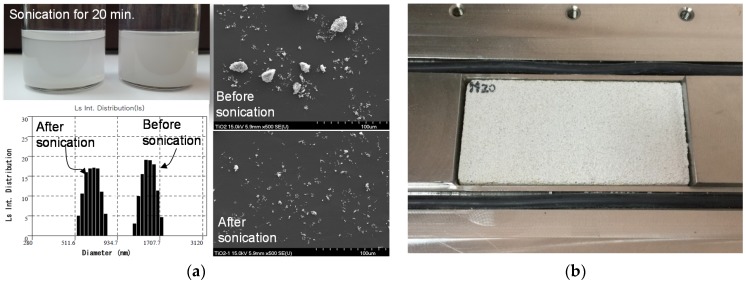
Specimen preparation: (**a**) sonicated aqueous TiO_2_ with particle size and distribution (dynamic light scattering (DLS)) and scanning electron microscopy (SEM) data; and (**b**) the specimen in the guide table in NO test machine.

Because NP-400 is cheaper than P-25, the mechanical and catalytic properties of cementitious materials with NP-400 deserve attention. We explored the compressive strengths and photocatalytic sensitivities (NO removal abilities) of such materials (cement paste and mortar) containing NP-400. To ensure that NP-400 inclusion did not compromise strength, NP-400 and P-25 at 0, 5, 10, and 20 wt % were mixed with cement pastes/mortars (5-cm cubes; Table 2). The water/cement ratio of cement pastes was changed between 0.50 and 0.625 (C samples) or between 0.5 and 0.588 (CP samples) for TiO_2_ contents ranging from 0% to 15% or 20% while for all mortar samples, the water/cement ratio was kept constant and equal to 0.50. If TiO_2_ was not able to develop pozzolanic or hydraulic activity, a decreasing trend would have been detected for cement pastes added with increasing TiO_2_ content. In contrast, for mortars, no significant variation in compressive strength values should have been expected. The mass per volume, 0–0.4 mg/mL (Table 2) was applied in this aqueous NP-400 dispersion with ultra-sonication while an ultra-sonication was not applied to aqueous P-25 solution. Dynamic light scattering (DLS) and scanning electron microscopy (SEM) confirmed that the NP-400 clusters were disaggregated (Figure 4), possibly increasing TiO_2_ concentrations on the top surfaces. These aqueous TiO_2_ dispersion was added after 5 min–dry mix of cement and sand. Consecutively, 3 more min for wet mix and 1 min for holding were applied before casting specimens. All specimens were de-molded after 1 day of curing at room temperature and placed in water for 27 days. Compressive strengths were measured using the 100-kN universal testing machine.

## 3. Results and Discussion

### 3.1. Mechanical Properties of Cement Pastes and Mortars

**Figure 5:**
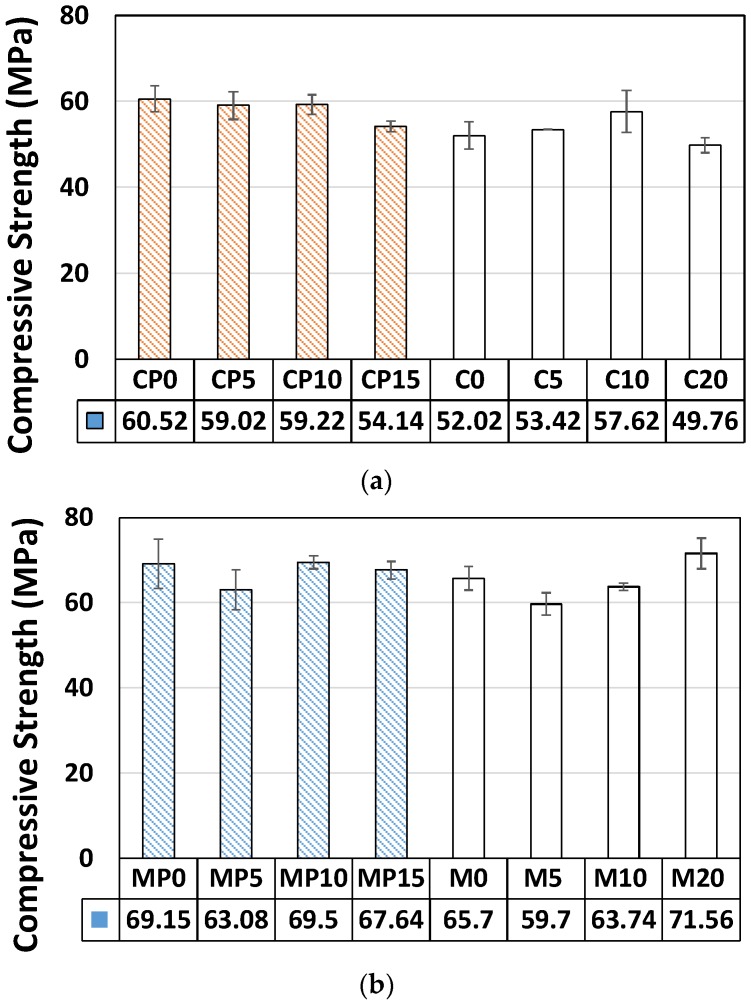
Compressive strengths of cementitious materials with different wt % values of P-25 and NP-400. (**a**) Cement paste. (**b**) Mortar. (C: cement paste, MP: mortar with P-25, M: mortar with NP-400).

We tested 48 specimens (Figure 5; CP: cement paste, and MP: mortar with P-25, C: cement paste, and M: mortar with NP-400). The wt % of NP-400 that referred to the cement content of pastes and to the sand content of mortars ranged from 0–20. For P-25, the wt % values were slightly different (0, 5, 10, and 15) because the P-25 volume at 20 wt % was too large to allow specimen casting. The apparent density of P-25 was 2.5-fold lower than that of NP-400. Figure 5 shows that the compressive strengths of cement paste has descending tendencies with increasing wt % of NP-400 and P-25. This indicates that the very small pozzolanic activities of TiO_2_ in the hydration process [12] may lead to these strength reductions. While the wt % of TiO_2_ referred to the sand content of mortar specimens, no significant change of strength was shown. Locally, the standard deviations (P-25 and NP-400 groups; Figure 5) were s_c-P25_ = 2.95 and s_c-NP400_ = 4.93 for cement paste specimens, and s_m-P25_ = 0.93 and s_m-NP400_ = 2.08 for mortar specimens. The deviations from the means were better in the P-25 groups, attributable to internal microvoids in the TiO_2_ clusters. P-25 has more voids than NP-400; loose bonding between molecules improves dispersion within cementitious materials. NP-400 is heavier than similar volumes of P-25 and cement powder, compromising dispersion within fresh cementitious materials. The changes in the standard deviations (Δs values) for both cement paste and mortar specimens containing P-25 and NP-400 were consistently about unity. Thus, future work should seek to control NP-400 fineness.

### 3.2. Nitrogen Oxide (NO) Removal by Cement Mixes Incorporating Titanium Dioxide

**Figure 6:**
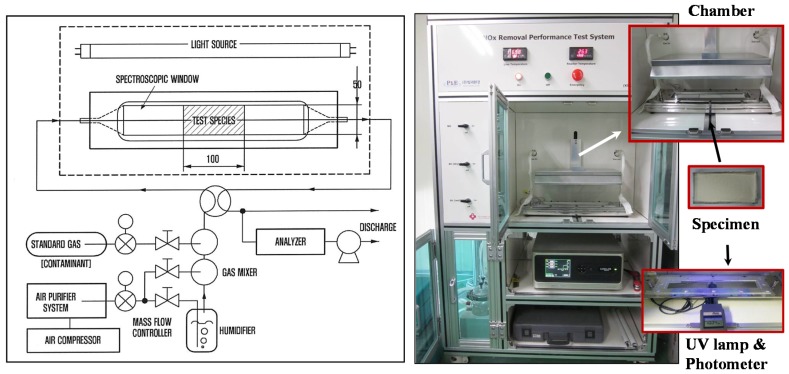
Apparatus meeting ISO 22197-1 specifications used to measure NO photocatalytic removal.

Rhee et al. [12] demonstrated that TiO_2_ (5 wt %) in mortar precipitated when cast; the TiO_2_ levels on the top and bottom surfaces of casts differed. Efforts to improve TiO_2_ dispersion in mortar or concrete (via the use of silica fumes or a high-range water reducer, or the addition of viscous agents, blast furnace slag, and/or foaming agents) barely affected TiO_2_ dispersion. Interestingly, even when TiO_2_ dispersion in mortar was enhanced, the NO reduction rates varied greatly by surface conditions. The surface void area affected NO adsorption and removal. However, even the creation of continuous low-frequency waves on a smooth surface did not affect the NO removal rate. We varied the TiO_2_ wt % values in cement paste and mortar, seeking to enhance surface photocatalytic reactions. We explored four different variables: (a) inclusion of sand or not; (b) the wt % of TiO_2_; (c) ultrasonication or not; and (d) compaction or not. The surface concentrations of TiO_2_ on cementitious materials should be maximized; this may be affected by the type of cement-based composite used (cement paste or mortar). We used 0, 5, 10, and 20 wt % cement (Table 2); each specimen had dimensions of 50 × 100 × 10 mm. Because NP-400 has a relatively large cluster size and is heavier than P-25, aqueous NP-400 solutions were subjected to ultrasonication at 750 W (20% duty cycle) for 20 min using a horn-type probe. Also, compaction during casting may alter the surface concentration of TiO_2_ via dynamic perturbation. Thus, specimens were placed on a plate-type vibrator operating at the maximum amplitude of 0.475 g at 20 Hz for 5 min. Eight specimens (16 surfaces) were analyzed in terms of NO removal under the same conditions. It is important to choose the right configuration of the reactor to improve the photocatalytic efficiency, allowing comparable and repeatable measurements. Even if the experimental setup includes other elements such as the light source, NO_x_ analyzer or the gas supplier, the core of the test setup is the photoreactor, which is responsible for an effective contact among photocatalyst, pollutants, water and light [30]. Understanding the above, we adopted ISO 22197-1 specification [31] in order to measure NO photocatalytic removal. All specimens were exposed to 1 ± 0.015 ppmv NO gas at a flow rate of 3.0 L/min, under UV of 10 W/m^2^ (Sankyo Denki 352-nm lamp, Hiratsuka, Japan) at 25 ± 2 °C and a relative humidity of 50 ± 5% for 2 h after removal of organic matter and impurities. Next, NO flowed in the dark for 30 min and the specimen was then UV-irradiated for 5 h; the NO removal rate was measured using an NO analyzer (CM2041, Casella, London, UK) and a photometer (HD9021, Delta Ohm, Padua, Italy) (Figure 6). The NO removal rate was the ratio of the initial NO concentration, *C_i_*, and that after 5 h of UV irradiation, *C_eq_* (Equation (1) and Table 3). The repeatability of the NO removal test for different specimens with the same wt % of NP-400 from the same batch showed no significant change, e.g., test #1: 43.3%, and test #2: 42.3% of removal rate for C20 specimen (back-face). These were done under the same test conditions as described at the earlier paragraph. Thereby, each test for the front/back-faces of all the specimen was performed once.

(1) $$\mathrm{NO}\;\mathrm{removal}\;\mathrm{rate}{\left(\%\right)}_{}=\left(\frac{C_{i}-C_{eq}}{C_{eq}}\right)\times 100=\frac{\Delta C}{C_{eq}}\times 100$$

**Figure 7:**
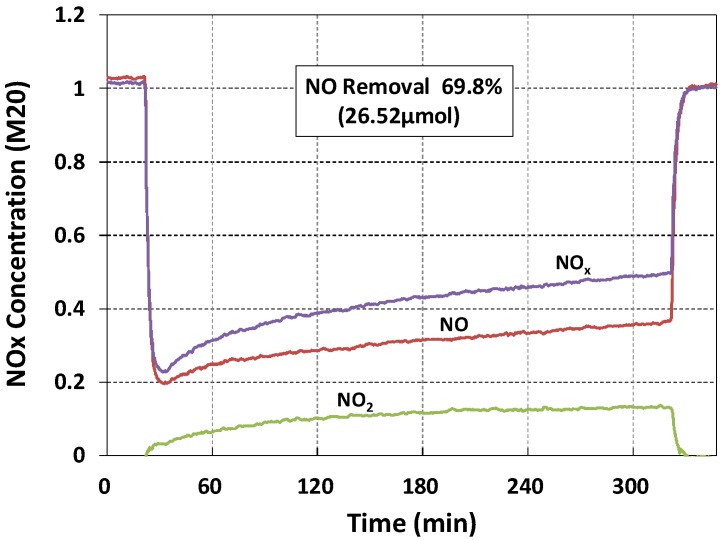
NO removal (ca. 70%) by M20.

**Figure 8:**
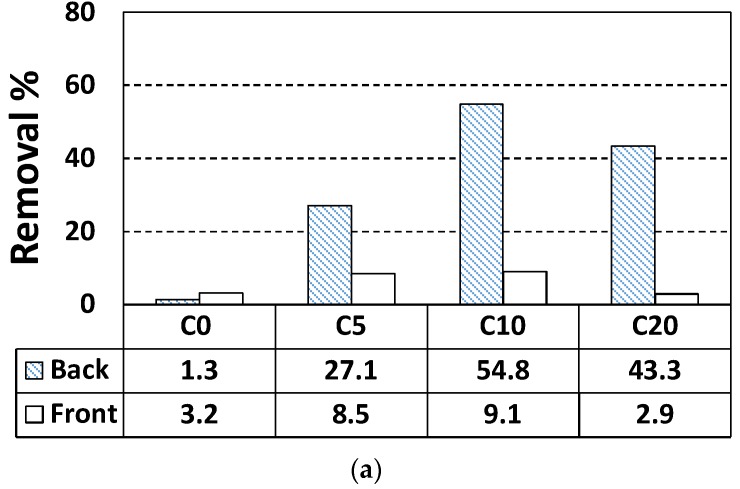

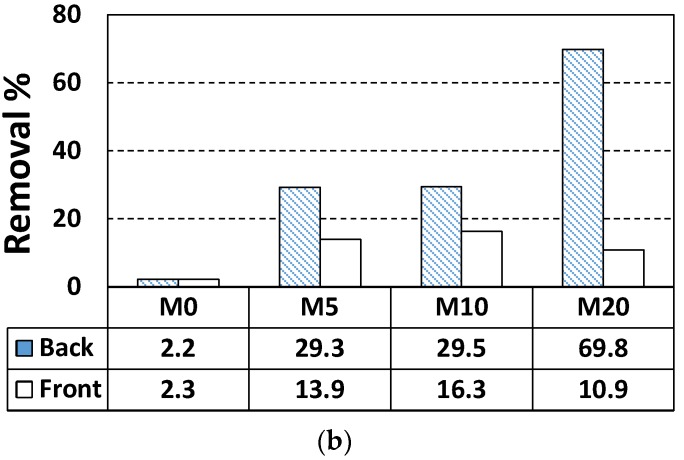
NO removal by cement paste and mortar: (**a**) cement paste; (**b**) mortar with NP-400.

NO removal was consistently better at the bottom of specimens (Table 3). M20 (mortar with 20 wt % NP-400) exhibited 69.8% NO removal (Figure 7). Removal by the top face was much lower. The brown line in Figure 7 indicates NO removal over the 5 h of the test. NO_2_ (another pollutant; green line) was adsorbed and penetrated the materials. The purple line shows the sum of these removals. Irregular staining by penetrating NO_2_ compromised the interaction of NO and TiO_2_, causing the NO removal line to slope upward. We will address only NO removal below. Such removal basically increased as the wt % of TiO_2_ rose (Figure 8). One another aspect is that C10 exhibits better NO removal efficiency rather than C20. This may be caused by a large NP-400 concentration inside the cement paste. The electron-hole recombination may occur when large amounts of NP-400 are present. Thus, the photocatalytic efficiency drops as a consequence of this recombination.

**Figure 9:**
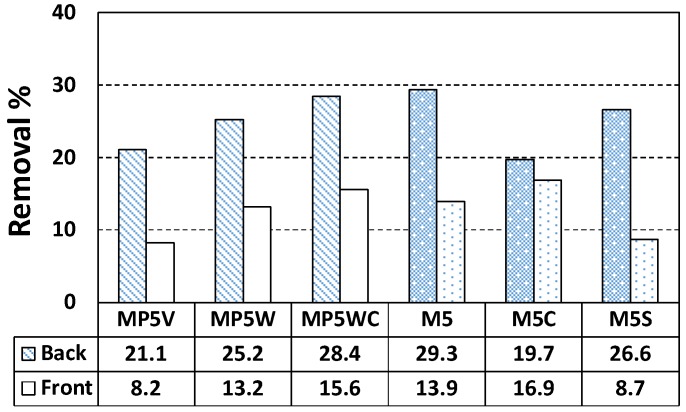
NO removal % by mortar (5 wt % TiO_2_) with and without ultra-sonication and dynamic compaction after casting (C: with compaction, S: with ultrasonication).

**Figure 10:**
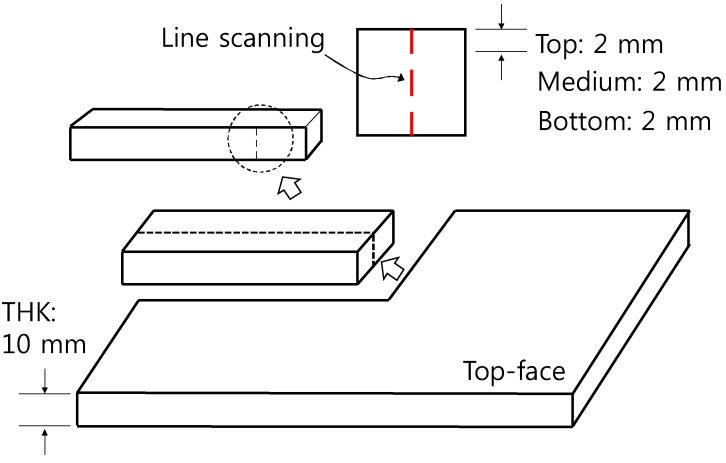
Specimen preparation for low vacuum (LV) SEM analyses: 25 × 10 × 10 mm.

Notably, although removal efficiency was best at the bottom faces of all specimens, the efficiency varied. Table 2 shows that the cement paste specimens (which lack fine aggregates) contained 2.33-fold more TiO_2_ than the mortar specimens; the cement paste specimens should thus remove NO better than the mortars. However, the reverse was true [e.g., C20 (TiO_2_-56 g, NO removal rate 43.3%) and M20 (TiO_2_-24 g, NO removal rate 69.8%)]. We explored this phenomenon. Since 20 wt % of TiO_2_ is a very large percentage, such a high percentages increases the final cost of the modified materials. Besides, some problems could arise linked to the mechanical performance if a portion of cement has been replaced with TiO_2_. The electron-hole recombination could have happened frequently when percentages of above 5 wt % of TiO_2_ were added. Thus, we set the TiO_2_ to 5 wt % for mortar and explored the NO removal rate in terms of wt % NP-400, wt % P-25, and dynamic compaction and ultrasonication status. M5 served as the control NP-400-containing mortar specimen. MP5 contained P-25, M5C and MP5WC were compacted and non-compacted specimens, and M5S was subjected to ultra-sonication during casting. MP5V and MP5W contained 2.4 and 6 g P-25, respectively. Figure 9 shows the NO removal rates of M5, MP5V, MP5W, M5C, MP5WC, and M5S. The bottom-face NO removal efficiencies were not affected by compaction or sonication. The control M5 specimen outperformed all test samples. However, by volume, MP5V (2.4 g, 21.1% TiO_2_) exhibited a better performance (in a non-proportional sense) than MP5W (6 g, 28.4% TiO_2_) by weight; P-25 may be a better photocatalyst than NP-400. Dynamic compaction improved the performances of the top faces of MP5WC and M5C. We further explored TiO_2_ distribution throughout the specimens (thickness 10 mm). Each specimen was bisected both vertically and horizontally and then cut horizontally once more through the center, and the final specimens were subjected to scanning electron microscopy with energy dispersive X-ray analysis (SEM–EDX), scanning from the top to the bottom (Figure 10). However, the thickness of 10 mm was excessive; we thus examined three 2-mm-long lines from the top, middle, and bottom (Figure 10).

**Figure 11:**
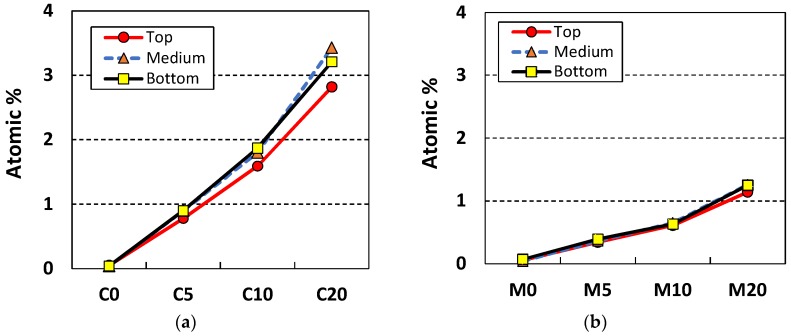

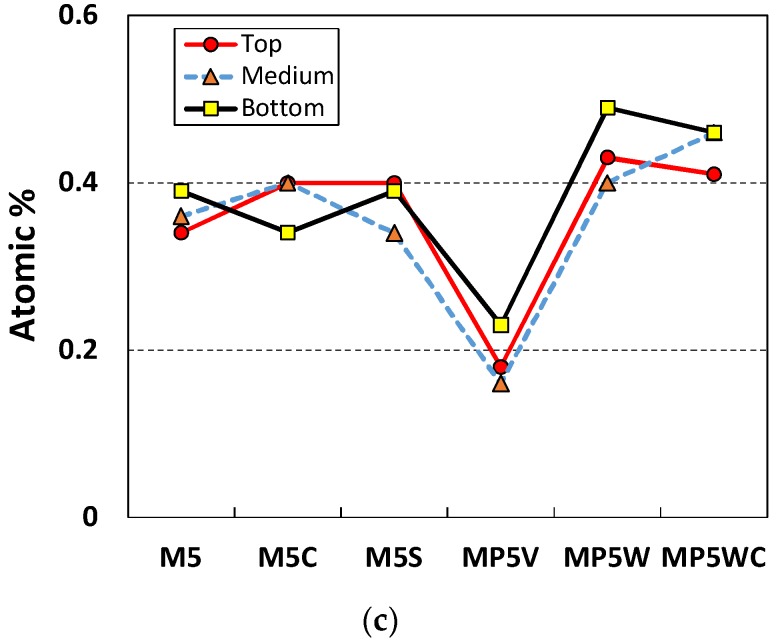
Atomic wt % values of slices of the 5 cm × 10 cm × 1 cm specimens: (**a**) cement paste specimens without compaction; (**b**) mortar specimens without compaction; (**c**) mortar specimens with compaction and sonication.

**Figure 12:**
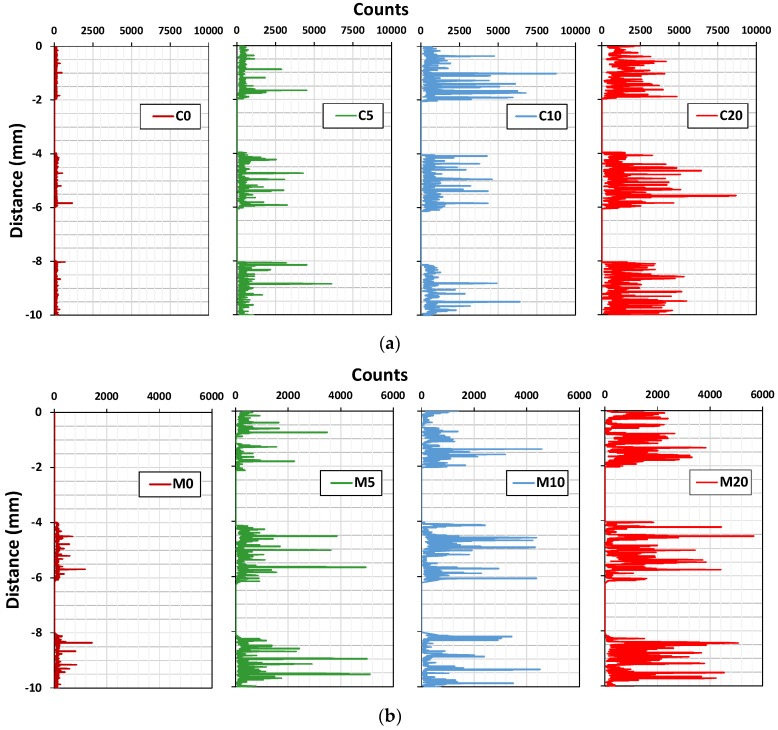
Ti counts on the top, middle, and bottom surfaces of sliced sections 10 mm in thickness (the line scans are at 2-mm intervals): (**a**) C-series samples, (**b**) M-series samples.

**Figure 13:**
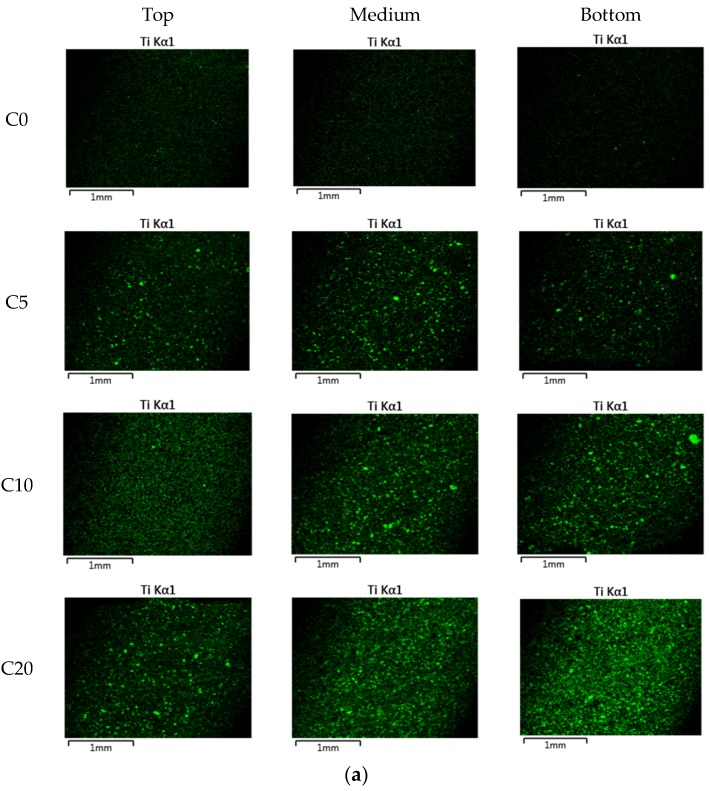

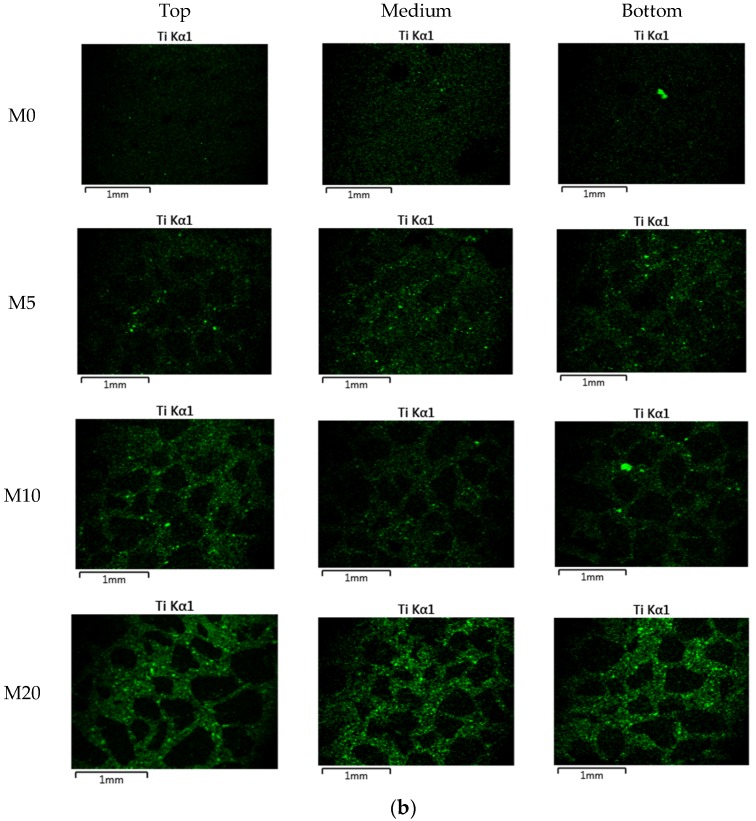
Ti distribution as revealed by LV-SEM with energy dispersive X-ray analysis (EDX) in cement paste and mortar specimens (green: Ti, measured in 2 mm-squares of the top, middle, and bottom faces): (**a**) C-series samples, (**b**) M-series samples.

**Figure 14:**
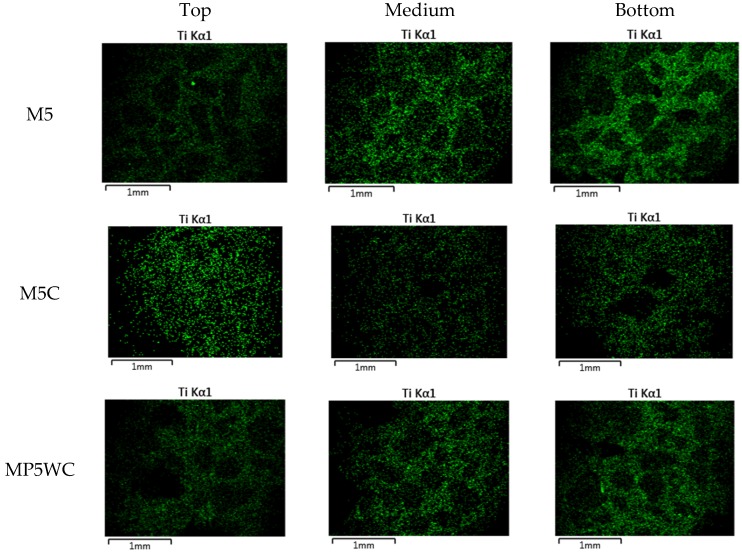
Ti distribution as revealed by LV-SEM/EDX for 5 wt % TiO_2_ mortar specimens subjected to dynamic compaction (Green: Ti, measured in 2 mm-squares of the top, middle, and bottom surfaces).

Figure 11 shows that content of Ti (wt %) measured at different depth of cement paste and mortar specimens containing NP-400. As the NP-400 levels rose from 0 to 20 wt %, the NP-400 concentrations increased in all sections. Figure 11a,b show the variations in NP-400 concentrations. Those of the top and bottom cement paste sections varied considerably (Figure 11a). The variations in the M5 series were minor, except for MP5V, which had a 2.5-fold lower level of NP-400 than the others. Figure 12 shows the line scanning results from the top, middle, and bottom of all specimens. The Ti intensity increased as the NP-400 wt % rose. The differences between the top and bottom sections were moderate; the Ti concentration was generally higher at the bottom face. Figure 13 and Figure 14 show the SEM–EDX mapping photographs. Green indicates Ti in 2 × 2 mm squares of the top, middle, and bottom faces, and the results confirm that NP-400 tends to sink when added to cement, fine aggregates, and water, because the true densities of P-25, NP-400, and cement powder were 3.41, 3.70, and 3.15 g/mL (Table 1); thus, NP-400 had the highest true density.

**Figure 15:**
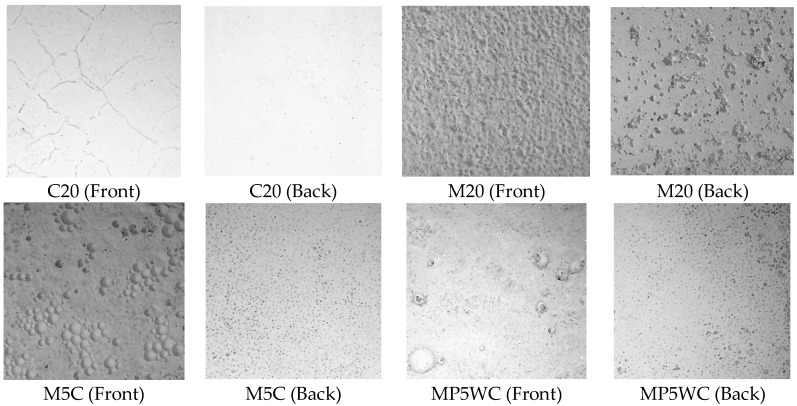
Front and back surfaces of selected specimens (25-mm squares).

Figure 14 shows the effects of dynamic compaction on the top surfaces, especially that of M5C. The surfaces exhibit many green dots. NO removal efficiency did not differ between the C- and M-series of NP-400 specimens, as discussed above. Although the NP-400 levels in mortars were 2.33-fold less than those in cement paste specimens, NO removal by mortars was much better than removal by cement pastes, probably because mortars have a greater photocatalytic surface area. Figure 15 shows the top and bottom faces of C20, M20, M5C, and MP5WC. M20 exhibited the best NO removal; the bottom surface area was greater than that of the top. In contrast, the bottom surface of C20 was smooth. This explains why the lower TiO_2_ concentration in M20 was actually better than the 2.33-fold larger level in C20. Dynamic compaction of M5C created many bubbles on the top surface (Figure 15), improving NO removal.

In summary, two major factors affect NO removal efficiency: TiO_2_ density and surface roughness. The former can be improved using higher incineration temperatures. However, the latter is paradoxical: higher surface roughness absorbs air pollutants more efficiently but also collects dust associated with staining, which blocks photocatalysis.

## 4. Conclusions

We evaluated how four different variables: (a) inclusion of sand or not; (b) the wt % of TiO_2_; (c) ultrasonication or not; and (d) compaction or not, affected NO removal by the type of cement-based composite used (cement paste or mortar) in accordance with the ISO 22197-1 standard (a 5-h test). Addition of NP-400 to 0, 5, 10, and 20 wt % did not significantly affect compressive strength. However, the compressive strengths of P-25 specimens were more consistent. The compressive strengths of cement paste have descending tendencies with increasing wt % of NP-400 and P-25. This indicates that the very small pozzolanic activities of TiO_2_ in the hydration process may lead these strength reductions. While the wt % of TiO_2_ referred to the sand content of mortar specimens, no significant change of strength was shown. The highest removal efficiency was ca. 70% by mortar with 20 wt % NP-400. Accessible surface roughness aid NP-400 action; such roughness readily adsorbs NO, given the large surface areas of the top and bottom faces. M20 exhibited the best NO removal efficiency and the bottom surface was larger than the top. Such removal basically increased as the wt % of TiO_2_ rose. However, C10 exhibits better NO removal efficiency than C20. This may be caused by a large NP-400 concentration inside the cement paste. The electron-hole recombination may occur when large amounts of NP-400 are present. Thus, the chance to combine with the calcium in cement pastes might be reduced due to excessive TiO_2_ concentration on the surface. SEM-EDX confirmed that NP-400 tended to sink when added to cement, fine aggregates, and water. The true densities of P-25, NP-400, and cement powder are 3.41, 3.70, and 3.15 g/mL. The relatively low apparent density of P-25 (compared to NP-400) creates a more bulky distribution inside cementitious materials. In sum, there were two main contributing factors to enhance the NO removal efficiency. One is the density of TiO_2_ and the other is the surface roughness. The former could be elaborated by applying higher incineration temperature in the kiln. However, the latter has a paradoxical aspect in that the greater surface roughness can absorb the air pollutants efficiently; at the same time, more dust/stain can sit on the surface. This dust-captured environment with high surface roughness can block the continuous chain of photocatalytic action. Also, a large amount of TiO_2_ addition would be affected by electron-hole recombination as well as some durability issues, especially for the freeze-and-thaw resistance of cement composites. Therefore, the trade off wt % of TiO_2_ should be selected by considering the strength, NO removal efficiency and durability concerns. Nevertheless, NP-400 could be a viable alternative to the definitive product, P-25.

## Figures and Tables

**Table 1:** TiO_2_ preparations mixed with cementitious materials.

Ingredients	Crystal Phase	Average Particle Size (nm)	Specific Surface Area (m^2^/g)	Apparent Density (g/mL)	True Density (g/mL)
NP-400	Anatase	26.6 (s = 8.52%) *	66.5	0.45	3.70
P-25	Rutile/Anatase	25.2 (s = 9.45%) *	54.9	0.18	3.41
Cement	Portland Type I	>44 μm	0.33	-	3.15

* s: standard deviation.

**Table 2:** Test specimens, wt % values with respect to cement content.

Sample Type	Label	Cement (g)	Sand (g)	Water (mL)	TiO_2_ (g)
Mortar (NP-400)	M0	120	240	60	0
	M5		234		6
	M10		228		12
	M20		216		24
Mortar (P-25)	MP0		240		0
	MP5		234		6
	MP10		228		12
	MP15		222		18
Cement paste (NP-400)	C0	280	-	140	0
	C5	266			14
	C10	252			28
	C20	224			56
Cement paste (P-25)	CP0	280			0
	CP5	266			14
	CP10	252			28
	CP15	238			56

**Table 3:** NO removal rates by cement paste (C) and mortar (M) (unit: μmol/(50 cm^2^∙5 h)).

Specimen Label	Face	NO Removal Rate (%)	NO Concentration Change, Δ*C* (μmol)	NO Initial Concentration, *C_i_* (μmol)
C0	Back	1.3	0.50	38.38
	Front	3.2	1.25	39.13
C5	Back	27.1	10.48	38.65
	Front	8.5	3.25	38.21
C10	Back	43.3	16.47	38.04
	Front	2.9	1.11	38.17
C20	Back	54.8	21.18	38.65
	Front	9.0	3.49	38.81
M0	Back	2.2	0.87	39.46
	Front	2.3	0.87	37.87
M5	Back	29.3	11.60	39.59
	Front	13.9	5.55	39.94
M10	Back	29.5	11.50	38.97
	Front	16.3	6.36	39.01
M20	Back	69.8	26.52	38.00
	Front	10.9	4.22	38.71
